# Proteomics on malignant pleural effusions reveals ERα loss in metastatic breast cancer associates with SGK1–NDRG1 deregulation

**DOI:** 10.1002/1878-0261.13540

**Published:** 2023-11-02

**Authors:** Isabel Mayayo‐Peralta, Donna O. Debets, Stefan Prekovic, Karianne Schuurman, Suzanne Beerthuijzen, Mathilde Almekinders, Joyce Sanders, Cathy B. Moelans, Sandra Saleiro, Jelle Wesseling, Paul J. van Diest, Rui Henrique, Carmen Jerónimo, Maarten Altelaar, Wilbert Zwart

**Affiliations:** ^1^ Division of Oncogenomics, Oncode Institute The Netherlands Cancer Institute Amsterdam The Netherlands; ^2^ Biomolecular Mass Spectrometry and Proteomics, Bijvoet Center for Biomolecular Research, Utrecht Institute for Pharmaceutical Sciences Utrecht University and Netherlands Proteomics Centre The Netherlands; ^3^ Department of Pathology The Netherlands Cancer Institute Amsterdam The Netherlands; ^4^ Department of Pathology University Medical Center Utrecht The Netherlands; ^5^ Lung Cancer Clinics Portuguese Oncology Institute of Porto Portugal; ^6^ Division of Molecular Pathology The Netherlands Cancer Institute Amsterdam The Netherlands; ^7^ Department of Pathology Leiden University Medical Center The Netherlands; ^8^ Cancer Biology and Epigenetics Group Research Center of the Portuguese Oncology Institute‐Porto Portugal; ^9^ Department of Pathology Portuguese Oncology Institute of Porto Portugal; ^10^ Department of Pathology and Molecular Immunology, Institute of Biomedical Sciences Abel Salazar (ICBAS) University of Porto Portugal; ^11^ Laboratory of Chemical Biology and Institute for Complex Molecular Systems, Department of Biomedical Engineering Eindhoven University of Technology The Netherlands

**Keywords:** breast cancer metastasis, NDRG1, oestrogen receptor, proteomics, receptor conversion, SGK1

## Abstract

Breast cancer (BCa) is a highly heterogeneous disease, with hormone receptor status being a key factor in patient prognostication and treatment decision‐making. The majority of primary tumours are positive for oestrogen receptor alpha (ERα), which plays a key role in tumorigenesis and disease progression, and represents the major target for treatment of BCa. However, around one‐third of patients with ERα‐positive BCa relapse and progress into the metastatic stage, with 20% of metastatic cases characterised by loss of ERα expression after endocrine treatment, known as ERα‐conversion. It remains unclear whether ERα‐converted cancers are biologically similar to bona fide ERα‐negative disease and which signalling cascades compensate for ERα loss and drive tumour progression. To better understand the biological changes that occur in metastatic BCa upon ERα loss, we performed (phospho)proteomics analysis of 47 malignant pleural effusions derived from 37 BCa patients, comparing ERα‐positive, ERα‐converted and ERα‐negative cases. Our data revealed that the loss of ERα‐dependency in this metastatic site leads to only a partial switch to an ERα‐negative molecular phenotype, with preservation of a luminal‐like proteomic landscape. Furthermore, we found evidence for decreased activity of several key kinases, including serum/glucocorticoid regulated kinase 1 (SGK1), in ERα‐converted metastases. Loss of SGK1 substrate phosphorylation may compensate for the loss of ERα‐dependency in advanced disease and exposes a potential therapeutic vulnerability that may be exploited in treating these patients.

## Graphical abstract

Breast cancer's hormone receptor status is pivotal in prognosis and treatment. Many tumours express oestrogen receptor alpha (ERα), which can be lost after treatment (ERα‐conversion). We studied 47 metastatic breast cancer samples—ERα‐positive, ERα‐converted and ERα‐negative. ERα‐converted tumours do not recapitulate ERα‐negative cancer; luminal‐like features persist. SGK1 kinase activity is reduced in ERα‐converted metastases, representing an interesting new therapeutic candidate.

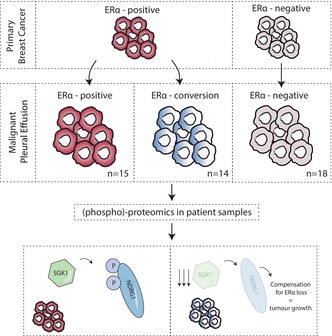

**Abbreviations**
AR: androgen receptor
BCa: breast cancer
CAA: chloroacetamide
DDA: data‐dependent acquisition
DIA: data‐independent acquisition
EDTA: ethylenediamine tetracetic acid
ERα: oestrogen receptor alpha
ERα‐C: oestrogen receptor alpha conversion
ERα‐N: oestrogen receptor alpha negative
ERα‐P: oestrogen receptor alpha positive
FFPE: formalin‐fixed paraffin embedded
GR: glucocorticoid receptor
H&E: haematoxylin‐and‐eosin
IHC: immunohistochemistry
KSEA: kinase‐substrate enrichment analyses
MPE: malignant pleural effusion
NDRG1: n‐myc downregulated gene 1
PCA: principle component analysis
PR: progesterone receptor
RTK: receptor tyrosine kinase
SDC: sodium deoxycholate
SGK1: serum/glucocorticoid regulated kinase 1
SHRs: steroid hormone receptors
TCEP: phosphinehydrochloride
TNBC: triple negative breast cancer

## 1 Introduction

Breast cancer (BCa) is a highly heterogeneous disease comprising distinct molecular subtypes and is the most commonly diagnosed cancer type in women worldwide [1, 2]. Approximately 70% of BCas are positive for the expression of Oestrogen Receptor alpha (ERα), which drives tumour growth and progression. Although endocrine therapies targeting the ERα signalling axis are highly effective, approximately 30% of BCa patients do not respond to treatment and develop metastatic disease [3]. Extensive efforts have been put into genomic profiling of advanced BCa, identifying different genetic alterations that drive endocrine‐therapy resistance, including mutations in the *ESR1* gene (encoding for ERα), Receptor Tyrosine Kinase (RTK) pathways, SWI/SNF complex members and other transcriptional modulators such as FOXA1 and CTCF [4, 5, 6, 7, 8, 9, 10]. Importantly, around 10–20% of advanced BCa cases are characterised by the loss of ERα expression, hereafter referred as ERα‐conversion (ERα‐C), which occurs as a consequence of prolonged exposure to endocrine therapies [4, 11, 12, 13]. ERα‐C is suggested to occur through epigenetic suppression mechanisms [14].

In contrast to extensive research on the biological features of ERα‐positive (ERα‐P) metastatic disease [4, 11], little is currently known about the implications of ERα‐C on disease progression and therapy response. Thus, an in‐depth characterisation of the molecular landscape of ERα‐C is needed. Due to loss of ERα expression, ERα‐C tumours are no longer sensitive to ERα‐targeting endocrine therapies and may depend on other signalling cascades to regulate cell proliferation. Therefore, these pathways may be exploited as potential new therapies for ERα‐C metastatic disease.

To gain better insights into the proteomic alterations in cellular signalling that occur upon ERα loss in metastatic BCa, we performed whole proteome and phospho‐proteome analyses of 47 metastatic BCa tumours obtained from 37 metastatic BCa patients, including 15 of ERα‐P, 14 ERα‐C and 18 ERα‐N (triple‐negative BCa cases). Strikingly, our data revealed that loss of ERα expression in advanced disease does not result in a complete switch to an ERα‐N molecular phenotype, as ERα‐C tumours retained luminal‐like features. These findings highlight the importance of in‐depth profiling of metastatic BCa tumours beyond subtyping by use of classical BCa biomarkers. Importantly, we found evidence for inactivation of several key kinases upon transition to ERα‐C. Particularly, loss of SGK1 activity and phosphorylation of its substrate NDRG1 may play a role in loss of ERα‐dependency during disease progression. These findings suggest that modulation of this pathway could be a promising strategy for future clinical interventions.

## 2 Materials and methods

### 2.1 Sample collection

Build‐up fluid in the pleural cavity was collected between November 2013 and July 2017, at the Netherlands Cancer Institute and at the Portuguese Oncology Institute, including a total of 47 MPEs (15 ERα‐P, 14 ERα‐C, 18 ERα‐N) from 37 patients. Samples were collected and processed immediately after drainage as described before [12]. In short, pleural fluid was centrifuged at 1600 g for 8 min, and erythrocytes were lysed, using the corresponding buffer (5 mm KHCO_3_, 75 mm NH_4_Cl, 400 μL 500 mm EDTA, up to 500 mL MilliQ, pH: 7.4) for 10 min at room temperature. Cells were then either resuspended using 10% DMSO solution and stored at −80 °C, or fixed with formalin and paraffin‐embedded for future immunohistochemistry stainings. This study was approved by the institutional review boards of the Netherlands Cancer Institute (CFMPB411) and of the Portuguese Oncology Institute of Porto, Portugal. Informed written consent was provided by all participants in the study. The conducted research adhered to all relevant guidelines and regulations. The study methodologies conformed to the standards set by the Declaration of Helsinki.

### 2.2 Tissue processing for immunohistochemistry

Immunostainings were processed on the Ventana Bechmark ULTRA autostainer (Roche) according to the manufacturer's recommendations with antibody detection using the OptiView DAB IHC Detection Kit (Roche Tissues Diagnostics). Briefly, formalin fixed paraffin embedded (FFPE) tissue sections of 3 μm thick were cut, deparaffinised, blocked, pretreated with the TRIS‐ethylenediamine tetracetic acid (EDTA)–boric acid pH 8.4 buffer Cell Conditioning 1 (Roche; CD4: 64 min, CD8: 24 min, CD20: 40 min and CD68: 24 min) and stained with monoclonal antibodies directed against CD4 (Cellmarque; #104R‐16, clone SP35, 1 : 20, incubation time 32 min), CD8 (Dako; #M7103, clone C8/144B, 1 : 100, incubation time 32 min), CD20 (Dako; #M755, clone L26, 1 : 400, incubation time 16 min) or CD68 (Roche; #790‐2931, clone KP1, ready to use, incubation time 32 min. Stainings for ERα were performed as previously described [13] An experienced pathologist assessed the percentage of tumour cells and the intensity of IHC staining in tumour cells. The scoring of the samples was done using the online platform Slidescore (www.slidescore.com).

### 2.3 Sample preparation for (phospho)proteomics

Samples were frozen and kept at −80 °C until cell lysis. Cell lysis was performed using SDC lysis buffer (1% sodium deoxycholate (SDC), 10 mm tris(2‐carboxyethyl) phosphinehydrochloride (TCEP), 40 mm chloroacetamide (CAA) and 100 mm TRIS, pH 8.0 supplemented with phosphatase inhibitors (PhosSTOP; Roche) and protease inhibitors (complete mini EDTA‐free; Roche) as described previously [14]. Proteins were digested using a two‐step digestion. First, proteins were digested for 2 h using LysC (Wako Chemicals Europe GmbH, Neuss, Germany) at 37 °C (enzyme to protein ratio 1 : 75). Next, trypsin (Sigma‐Aldrich) was added in an enzyme to protein ratio 1 : 50 and digestion was performed overnight at 37 °C. Next, samples were acidified, and desalted using Oasis HLB μElution (Waters, Etten‐Leur, The Netherlands). Samples were aliquoted for full proteome analysis (40 μg), in‐house build library (30 μg), phosphopeptide enrichment (200 μg). Phosphopeptides were enriched using the AssayMAP Bravo Platform (Agilent Technologies) using Fe(III)‐NTA cartridges (Agilent Technologies) as described previously [14]. Samples were dried and stored at −20 °C until LC–MS analysis.

### 2.4 Library build: high pH fractionation

A peptide‐fragment library was built in‐house to match against the acquired DIA spectra. Therefore, 10 μg of each sample was pooled for the full peptide library and 20 μg of each sample was pooled for the phosphopeptide library. Pooled samples were fractionated on a high‐pH reversed‐phase C18 column (Kinetex 5u Evo C18 100A, 150 × 2.1 mm; Phenomenex) coupled to an Agilent 1100 series HPLC over a 50 min gradient. Fractions were concatenated to 20 fractions for the proteome library and seven fractions for the phosphoproteome library. Samples were dried down and stored at −20 °C. Full peptide library samples were read for MS analysis. The phospho‐peptide library samples were enriched for phosphorylated peptides as described previously [14].

### 2.5 LC–MS/MS analysis

Two different LC–MS/MS analysis were performed. First, a data‐dependent analysis (DDA) was performed on the fractionated samples to build a library. Second, sample spectra were acquired using data‐independent analysis (DIA). Samples were reconstituted in 10% FA, including 1× iRT (Biognosys) for retention time alignment. Peptides were separated prior to MS/MS analysis on an Agilent 1290 Infinity System (Agilent Technologies), directly coupled to a Q Exactive HF (Thermofischer Scientific). Peptides were trapped on a trap column (100 μm i.d. by 2 cm, packed with C18 resin) at a flowrate of 5 μL·min^−1^ using buffer A (0.1% FA). Peptides were subsequently pushed onto the analytical column (75 μm i.d. by 50 cm, packed with 2.7 μm Poroshell C18 material) and eluted by increasing buffer B (80% ACN 0.1% FA) from 13% to 44% during 120 min for the full peptide measurements. Gradient was set from 8% to 32% during 120 min for the phopshopeptides. The subsequent MS analysis differed for the library build and the sample measurement. MS settings for the DDA library were as follows: MS1 resolution was set to 60 000 with AGC target of 3e6 and scan range from 395 to 1005 *m*/*z*. The top 15 precursors were chosen for fragmentation and MS2 measurement. MS2 resolution was set to 30 000, AGC target to 1e5 and max injection time to 65 ms. Normalised collision energy was set to 27. MS settings for DIA were as follows: MS1 resolution was set to 60 000, AGC target was 3e6 and maximum injection time was 65 milliseconds and scan range from 395 to 1005 *m*/*z*. Fragment spectra were measured at a 30 000 resolution, AGC target of 1e6 and automatic maximum injection time. Loop count was 30 and isolation window 20 *m*/*z*, ranging from 410 to 990 *m*/*z*. Normalised collision energy was set to 27.

### 2.6 Data processing

DDA MS files were searched using Mascot search engine and the human Swissprot database in Proteome Discoverer (version 2.3.0.522). Trypsin was set as cleavage enzyme and up to two missed cleavages were allowed. Oxidation (M) and Acetylation (N‐terminus) were set as dynamic modifications. Carbamidomethyl (C) was set as a fixed modification. FDR for peptides and PSMs was set to 1%. For the phosphopeptide search, phosphorylation (STY) was added as a dynamic modification and up to three missed cleavages were allowed. Minimum site probability for the phosphosite localisation was set to 75%.

The acquired MSF‐files from the P.D. search were used to build a library in Skyline. The cut‐off score was set to 0.99 and ion match tolerance to 0.05 *m*/*z*. The six most intense product ions were picked (with a minimum of four transitions per precursor) and only scans within 5 min of the predicted RT were included. Peptide length was set from 7 to 36 amino acids. A minimum of two peptides per protein was set. Included structural modifications were: carbamidomethyl (C), Oxidation (M), Acetyl (N‐term) and for the phospho analysis phospho (STY). Decoy peptides were generated by sequence shuffle.

Raw DIA MS files were searched against the acquired library using Skyline and subsequently mPROPHET scoring was used to discern true hits from decoys [15]. A *q*‐value cut‐off of 0.01 was used. Quantification was performed by integration of the chromatographic peak, using only high‐quality features. Data were Log_2_‐transformed and normalisation was performed by median subtraction. Missing values were imputed using missforest imputation method [16]. Heatmaps were generated in R using Euclidean distance on *z*‐scored data. Statistical testing was performed in r. All data were plotted using ggplot2 (v.3.3.5) or pheatmap (v.1.0.12).

### 2.7 Kinase substrate enrichment analyses

Kinase substrate enrichment analyses (KSEA) were performed to learn about changes in activity of a specific kinase by interrogating differential phosphorylation of all kinases' substrates [17]. kseaapp (v.0.99.0) was used to perform the analyses.

### 2.8 Genetic dependency analyses

The dependency data used in this study was obtained from DepMap 21Q4 public (https://depmap.org/portal/). Dependency data for *NDRG1*, *RB* and *OCA‐2* genes across BCa cell lines were downloaded. All data were plotted using ggplot2 (v.3.3.5), ggpubr (v.0.4.0) and pupillometryr (v.0.0.4).

### 2.9 Gene set over‐representation analyses

In order to carry out gene set over‐representation analyses, we made use of the GSEA browser and used C2 curated pathways. The analysis was carried on differentially expressed proteins (*P*‐value < 0.05, log_2_difference > 1.5 or < −1.5) between ERα‐C and ERα‐P samples.

## 3 Results

### 3.1 Design of the study: malignant pleural effusions from advanced metastatic BCa

**Fig. 1:**
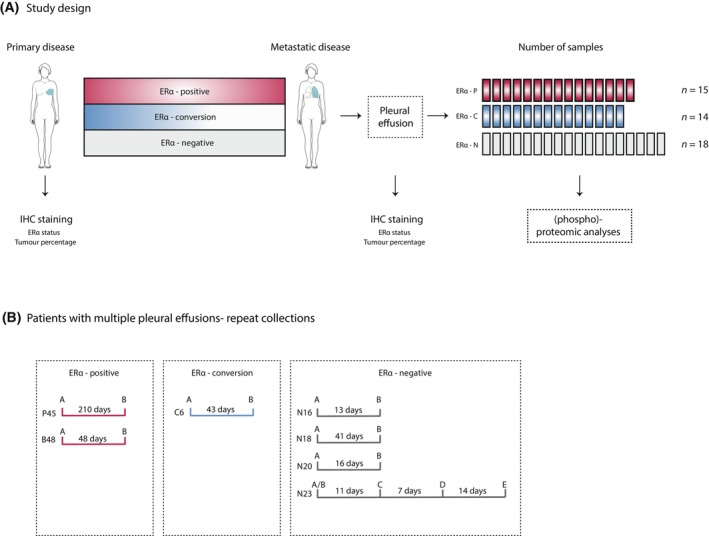
Study design and phospho‐proteomics data collection. (A) Study design. Primary and metastatic breast cancer were collected and stained for tumour cell percentage and Oestrogen Receptor alpha (ERα) status. Malignant pleural effusion samples were processed for total and phospho‐proteomics. (B) Patients with multiple pleural effusions, constituting repeat sample collections. Seven patients had samples collected at different time points. Time elapsed between collections is depicted in days.

To gain insights into BCa disease progression and endocrine treatment resistance due to loss of ERα expression, we performed proteomics and phospho‐proteomics in a cohort of patients with metastatic BCa disease. All advanced BCa tumours included in this study metastasized to the pleural cavity, resulting in malignant pleural effusions (MPEs). As palliative care, MPEs were drained out to facilitate the patient's breathing and improve quality‐of‐life in this highly progressive phase of the disease. MPEs were collected by centrifugation (see Section 2) and used for this study (Fig. 1A). Patients who were included in this study are detailed in Table S1. The study comprised three distinct group of patients: ERα‐positive (ERα‐P) (*n* = 15), ERα‐conversion (ERα‐C) (*n* = 14) and ERα‐negative (ERα‐N) (*n* = 18) cases. ERα‐P and ERα‐C were characterised by expression of ERα (50–100% ERα‐positive tumour‐cells) in primary disease, and by maintenance (5–100% ERα‐positive tumour cells, average = 58%) or loss (< 1% ERα‐positive cells) of ERα‐expression, respectively, in metastatic disease (Table S1). In addition, as a control we used samples from ERα‐N, which had no ERα expression throughout the course of the disease. In total, we collected 47 MPE samples, derived from 37 BCa patients. For seven patients, multiple MPEs were collected over a course of time (Fig. 1B). For six of these patients (two ERα‐P, one ERα‐C, three ERα‐N), two tumour samples were collected, over a period ranging from 2 weeks to 10 months; these are referred to as repeat collections. For one patient (ERα‐N), the data set contained five repeat collections, obtained over a period of 4 weeks (Fig. 1B). For all samples, Haematoxylin‐and‐Eosin (H&E) staining as well as Immunohistochemistry (IHC) for ERα were performed to enable assessment of tumour percentage (ranging between 35% and 90% in all samples) and status of ERα, progesterone receptor (PR) and HER2 (Table S1). In addition, information of all treatments prescribed in the adjuvant setting, treatment at time of pleural effusion, ILC/IDC status and interval time between primary tumour and disease progression and as well from progression to date of pleural effusion, is provided (Table S1). On these well‐annotated patient samples, we performed whole proteome and phospho‐proteome analyses.

### 3.2 Characterisation of proteomic data and exclusion of samples with immune cell infiltration

For (phospho)proteomic analyses, MPE cells were lysed, proteins were extracted and digested. The generated peptides were analysed by LC–MS/MS using Data‐Independent Acquisition (DIA). The acquired DIA files were matched against an in‐house built library. This library was generated using Data‐Dependent Acquisition (DDA) of pooled and fractionated samples. For the phosphorylation analysis, phospho‐enrichment was performed prior to LC–MS/MS analysis. An overview of the workflow is depicted in Fig. S1A. We obtained whole proteome and phospho‐proteome coverage of all patient samples, with a total of 6440 proteins and 10 772 phospho‐peptides quantified across all tumours (Fig. S1B,C).

Malignant pleural effusions are cell suspensions that contain multiple cell types, including not only metastatic BCa cells, but also reactive mesothelial cells and different types of immune cells [18]. Since the goal of this study was to gain insights into the molecular signature of metastatic tumour cells, we wanted to first ensure that the non‐tumour component of MPEs would not influence our downstream (phospho)proteomic analyses. In order to achieve this, we first performed unsupervised hierarchical clustering of all proteomic samples and found that most samples with a low tumour cell percentage cluster together, therefore suggesting similarity in their proteomic landscapes (Fig. S2A). Furthermore, principal component analysis (PCA) showed clustering of most of these same samples in the second component (Fig. S2B), indicating that the cell‐type composition affected the general proteomic profile and consequently drove sample clustering. In addition, the samples with low tumour cell content displayed strong enrichment of immune cell signature proteins (ImSIg database) [19], implying high immune cell infiltration (Fig. S2C,D). Based on these observations, eight samples—including three ERα‐P (P43/P35/P50), four ERα‐C (C5/C6B/C11/C12) and one ERα‐N (N30)—with an immune cell dominated proteomic signal and with low tumour cell content were excluded for further analysis (Fig. S2C,D). Moreover, we performed IHC analyses for immune markers, including CD4, CD8, CD20, CD68, and observed that samples excluded from the analyses were significantly enriched for CD4‐infiltrating cells (Fig. S2E). With this, we confirmed that there was an enrichment of infiltrating immune cells in the excluded samples which could have had an impact on downstream analyses of the (phospho)proteomic data sets.

### 3.3 Repeat collections from the same patient reveal patient‐specific proteomic signatures that remain stable over time

**Fig. 2:**
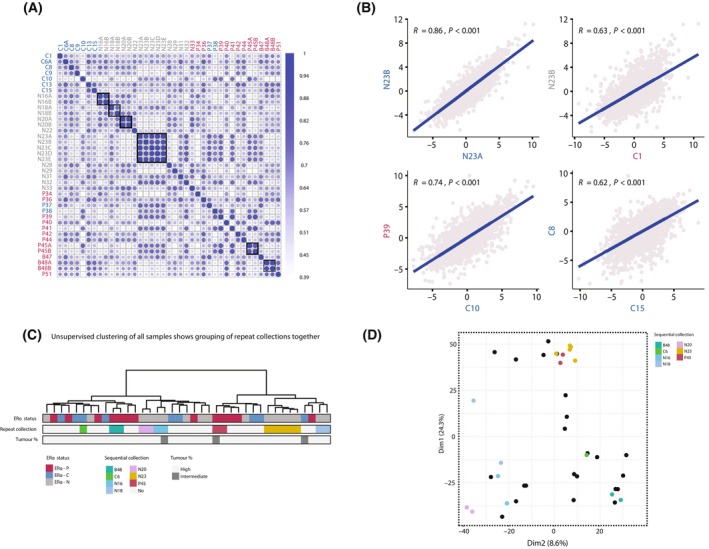
Repeat MPE collections from the same patient revealed patient‐specific proteome landscapes. (A) Correlation plot of all samples on proteomics data. Repeat collections are highlighted in the black boxes. Patient identifiers are coloured by Oestrogen Receptor alpha (ERα)‐status. (B) Correlation plots of individual patients demonstrating a higher level of correlation for samples from the same patient, relative to inter‐patient analyses. Pearson correlation is provided. (C) Unsupervised hierarchical clustering of all proteins in the data set grouped samples derived from the same patients together. The column colour bars indicate the patient group (ERα‐C (converted), ERα‐P (positive), ERα‐N (negative)), whether samples are repeat collections and tumour percentage. (D) Principle Component Analysis plot, coloured by repeat collection. Samples from the same patient cluster together.

We next assessed whether the proteomic profile of advanced BCa tumours changed over time during disease progression. To do this, we included repeat MPEs from the same patient that were collected over time (Fig. 1B). Interestingly, the proteomic profile of these metastatic tumours was highly reproducible; each patient displayed a patient‐specific proteomic signature that remained stable over time between sample collections and differed from other individuals (Fig. 2A, Fig. S3A). Importantly, we observed that repeat collections of the same patient were more similar as compared to samples between different patients (Fig. 2A,B). Moreover, the high correlation of repeat collections of the same patient was also illustrated by tight grouping of patients following unsupervised clustering analysis of the total proteomics data set (Fig. 2C), as well as grouping in PCA space (Fig. 2D, Fig. S3B).

The same observations were made in the phospho‐proteomic landscape (Fig. S3C). These findings suggest that MPEs from BCa remains stable over time with a limited subclonal selection. However, repeat collections were isolated over short periods of time, suggesting that time may have not been sufficient for enrichment of different of cell populations by clonal selection under treatment pressure.

### 3.4 A subset of ERα‐C tumours retains a proteomic signature similar to ERα‐P tumours

**Fig. 3:**
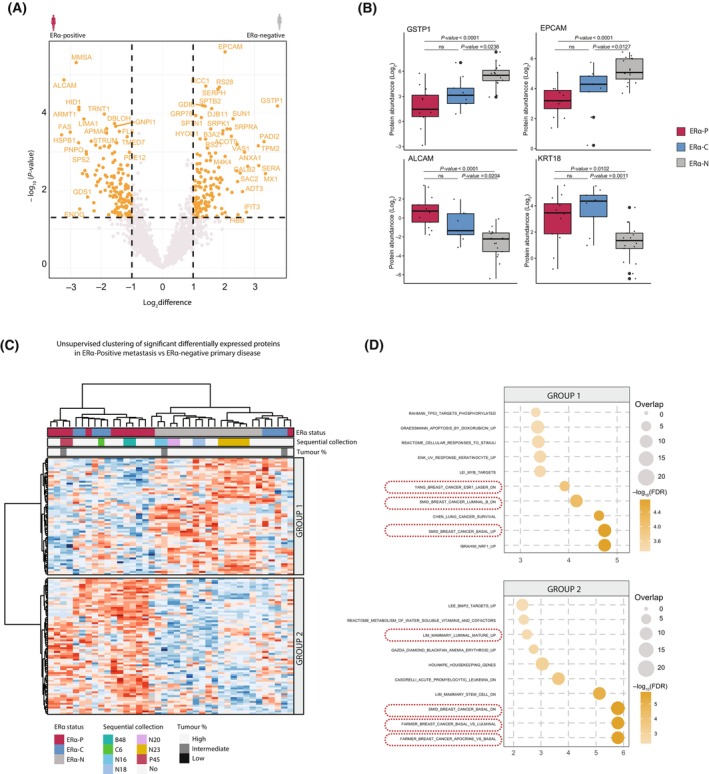
ERα‐C metastatic tumours retained ERα‐P proteomic signature. (A) Volcano‐plot of −Log_10_(*P*‐value) and Log_2_ difference between ERα‐N (negative) compared to ERα‐P (positive) metastatic breast cancer samples. Proteins coloured in orange were considered significantly changing (*P*‐value < 0.05 and Log_2_ difference > 1). (B) Boxplots of significantly changing proteins per ERα‐status. One‐way ANOVA test was used. *P*‐values are depicted. (C) Unsupervised hierarchical clustering of significantly differentially expressed proteins (*P*‐value < 0.05 and Log_2_ difference > 1.5). Unsupervised hierarchical clustering of all proteins in the data set grouped samples derived from the same patients together. The column colour bars indicate the patient group (ERα‐C, ERα‐P, ERα‐N), whether samples are repeat collections and tumour percentage. (D) Gene‐set overrepresentation analyses, proteins from C (group 1 and group 2) were used.

We next aimed to identify proteomic differences between metastatic tumours, based on ERα status. First, we performed a quantitative comparison of protein expression of ERα‐P and ERα‐N metastatic tumours. Our analyses revealed 288 differentially expressed proteins (*P*‐value < 0.05, log_2_difference > 1 or < −1) between the two groups (Fig. 3A). Notably, we found that proteins involved in triple‐negative breast cancer (TNBC), such as GSTP1 and EPCAM [20, 21], were enriched in ERα‐N metastatic tumours, while proteins typically upregulated in ERα‐P disease, such as ALCAM and KRT18 [22], were enriched in ERα‐P as well as the ERα‐C metastatic tumours (Fig. 3A,B). Interestingly, we found that the expression of those proteins in the ERα‐C group was similar to the ERα‐P samples, deviating from the ERα‐N samples (Fig. 3B). These findings suggest that despite the loss of ERα expression, ERα‐C metastatic tumours may have a proteomic landscape like the one of ERα‐P metastatic tumours, and not that of ERα‐N tumours.

The above‐mentioned data indicate that on proteomic level ERα‐P tumours resemble ERα‐C tumours. To determine whether these observations are also consistent on a global level, we performed unsupervised clustering of differentially expressed proteins (*P*‐value < 0.05, log_2_difference > 1.5 or < −1.5) and we observed that ERα‐N patients clustered away from ERα‐P/C patients (Fig. 3C). Importantly, we observed that there were two major groups of differentially expressed proteins, comprising a group of proteins mainly expressed in ERα‐N/C patients (group 1) and a group of proteins that are more abundantly expressed in ERα‐P/C patients (group 2) (Fig. 3C). Interestingly, by performing over‐representation analyses we observed that proteins from group 1 belonged to pathways expressed in basal BCa (Fig. 3D). On the other hand, the same analyses on proteins from group 2 demonstrated enrichment of proteins contributing to luminal BCa pathways (Fig. 3D). Overall, our data suggest that a subset of ERα‐C tumours has a similar proteomic landscape to ERα‐P rather than ERα‐N, even though ERα‐C tumours have lost the expression of ERα during the progression of the disease. These findings expand our understanding of the molecular mechanisms of BCa metastasis.

### 3.5 Decrease in kinase activity in ERα‐converted cancers contributes to tumour progression

**Fig. 4:**
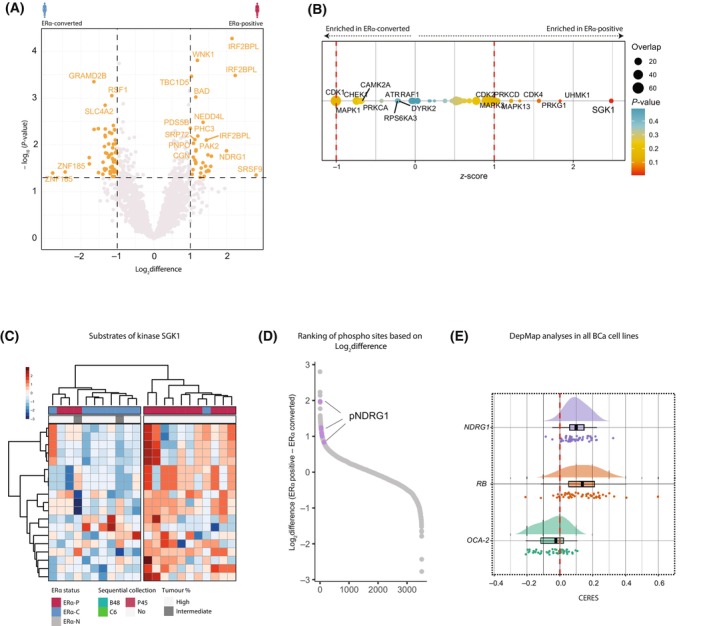
Decrease in kinase activity in ERα‐converted cancers contributes to tumour progression. (A) Phospho‐proteomics data. Volcano‐plot of −Log_10_(*P*‐value) and Log_2_ difference between ERα‐C (converted) compared to ERα‐P (positive) metastatic breast cancer samples. Phospho‐proteins coloured in orange were considered significantly changing (*P*‐value < 0.05 and Log_2_ difference > 1). (B) Kinase substrate enrichment analyses (KSEA) on differentially phospho‐peptides between ERα‐C and ERα‐P metastatic breast cancer samples. (C) Unsupervised hierarchical clustering of phosphorylation substrates of SGK1 kinase. The column colour bars indicate the patient group (ERα‐C, ERα‐P), and tumour percentage. (D) Phospho‐peptides ranked based on Log_2_difference between ERα‐P and ERα‐C samples. All the phospho‐NDRG1 peptides are highlighted in purple. (E) DepMap analyses of NDRG1 essentiality in breast cancer cells. Expression of RB (tumour suppressor) or OCA‐2 (no proliferation effects) are used as control. Essentiality scores are plotted (CERES).

In order to understand whether alternative signalling cascades compensate for the loss of ERα in ERα‐C tumours, we first compared the phospho‐proteomic profiles of ERα‐P and ERα‐C samples and identified 86 phospho‐sites differentially expressed (*P*‐value < 0.05, log_2_difference > 1 or < −1) (Fig. 4A, Fig. S4A). We next wanted to further investigate the biological consequences of changes in the phospho‐proteomic landscape. To address this, we performed kinase‐substrate enrichment analyses (KSEA) [17], which enabled identification of differentially activated kinases between patient groups. We observed that ERα‐P were enriched for activity of different kinases including SGK1, UHKM1 and PRKG1 kinases (Fig. 4B). The latter suggests that pathways regulated by the aforementioned kinases were downregulated in ERα‐C samples. As the SGK1 was the top enriched kinase, we specifically focused on interrogating differential phosphorylation on SGK1‐downstream targets. For this, we performed unsupervised hierarchical clustering of ERα‐P and ERα‐C of all identified substrates of the kinase SGK1 (Fig. 4C). Interestingly, we found that ERα‐C samples cluster away from ERα‐P samples, exhibiting a general loss of phosphorylation for all identified SGK1 targets (Fig. 4C). Next, we ranked all differentially phosphorylated sites between ERα‐P and ERα‐C and observed that the phosphorylation sites of a well‐described SGK1 target, NDRG1, were among the most highly differentially phosphorylated sites (Fig. 4D). Indeed, when correlating individual patients from either ERα‐C or ERα‐P groups, we observed an enrichment for phosphorylation of NDRG1 in ERα‐P samples (Fig. S4B). Downregulation of activity of the SGK1‐NDRG1 signalling has been reported to be involved in tumour metastasis and invasion [23]. These data suggest that ERα‐C samples lose activity of NDRG1, subsequently leading to downstream activation of tumour cell survival cascades. In line with this, we made use of the DepMap database to study the essentiality of NDRG1 in proliferation of BCa cells (Fig. 4E). Importantly, we observed that depletion of NDRG1 resulted in a proliferation advantage of BCa cells, comparable to the effect observed when perturbing expression of the well‐known tumour suppressor Rb1, indicating a role of NDRG1 as a tumour suppressor in BCa (Fig. 4E). Further follow‐up studies should confirm the role of NDRG1 in ERα‐C disease. Based on our findings, we propose that re‐activation of the SGK1‐NDRG1 axis in ERα‐C samples may be exploited as a new therapeutic target in ERα‐C patients.

## 4 Discussion

Endocrine resistance poses a major clinical problem in the treatment of BCa, and substantial efforts have been made to identify players responsible for disease progression and metastasis [11]. Although acquisition of specific genomic features in endocrine resistant metastatic BCa have been extensively reported [4, 9, 10, 24, 25, 26], including mutations of the *ESR1* gene, few studies have interrogated the implications of ERα loss in the metastatic setting, which accounts for around 20% of all endocrine‐resistant BCa cases [4, 27, 28]. In this study, we aimed to gain insights into the proteomic features of advanced BCa upon loss of ERα‐expression, to leverage discovery of potential therapeutic vulnerabilities for these tumours. To achieve this, we conducted proteomic and phospho‐proteomic analyses of metastatic BCa tumours, in relation to loss of ERα.

First, our study demonstrates that multiple tumour collections from the same individual display highly similar proteomic profiles, indicating that these metastatic lesions likely consist of a relatively stable tumour cell population that remains largely unaltered throughout the course of the disease. It is important to note that the limitations of the technology used and the short time intervals (from 2 weeks to 7 months, see Table S1) between the collections MPEs may account for the absence of tumour subpopulations being enriched during treatment due to clonal selection under therapy pressure. In line with this, a previous study [29] used orthogonal approaches such as single‐cell and bulk RNA‐seq, as well as DNA‐seq to track subclonal evolution in metastatic BCa patient samples. In this study, due to longer period times (2–15 years; Table S1) between MPE collection, they were able to identify subclones that became more prominent over time and investigated the efficacy of drugs targeting the enriched subclones.

We demonstrated that tumours that lose ERα‐expression during disease progression, retained a proteomic signature more similar to ERα‐P metastases (derived from ERα‐P primary disease), as opposed to ERα‐N metastases (derived from ERα‐N primary disease). Our proteomics data further suggest that part of the ERα‐related translational programme is still active in some of ERα‐C tumours since ERα‐target proteins did not diminish to levels of ERα‐N tumours. However, ERα‐targeting therapies would not be efficient in these patients due to the lack of ERα expression, which suggests that targeting downstream targets may be a potential therapeutic route. We hypothesise that the similar proteomic landscape between ERα‐P and ERα‐C tumours could be due to activity of other steroid hormone receptors (SHRs), such as Androgen Receptor (AR), PR, or Glucocorticoid Receptor (GR), now compensating for loss of ERα action. Previous research has shown that these SHRs are able to bind to similar areas in the genome as ERα and consequently induce similar transcriptional programmes, both *in vitro* and in patient samples [30, 31, 32, 33, 34, 35, 36]. In the absence of ERα‐expression, these ERα‐binding sites could be hijacked by AR, PR, or GR to maintain expression of genes contributing to tumour cell survival [30, 31, 32, 33, 34, 35, 36]. Due to the technical limitations of mass spectrometry, no peptides were detectable in relation to the mentioned SHRs. Consequently, further mechanistic investigations are imperative to formally test this hypothesis.

We identified the activity of kinase SGK1 to be enriched in ERα‐P samples when compared to ERα‐C samples. In line with this, we observed loss of phosphorylation of NDRG1, a known target of SGK1, in ERα‐C samples [37]. NDRG1 has been described as a metastasis suppressor across different tumour types [23]. The phosphorylation of NDRG1 by SGK1 has been shown to be crucial to halt downstream oncogenic pathways [38]. When active, NDRG1 results in inhibition of cell proliferation, migration and metastasis [23]. In the scope of our investigation, centred around the analysis of notably aggressive samples, it is plausible to propose the hypothesis that the diminished functionality of SGK1 in ERα‐C leads to the reduction in NDRG1 phosphorylation in a context‐dependent manner. This absence, in turn, could facilitate the maintenance of cellular proliferation pathways in ERα‐C samples, while alternative pathways might govern tumour growth in the other two groups. Through *in silico* analyses, we observed that loss of NDRG1 expression resulted in a growth advantage across a panel of BCa cell lines. Future mechanistic studies are warranted to explore the context‐specific characteristics underlying the disruption of the SGK1‐NDRG1 axis. Our findings could suggest that modulation of NDRG1 expression might be a novel therapeutic avenue for ERα‐C samples. Cellular stress as a result of iron depletion, among others, leads to the increase of NDRG1 expression [39]. Different studies used iron‐binding agents to upregulate the expression of NDRG1, resulting in strong antitumour efficacy, which could be exploited for the treatment of ERα‐C patients [23, 40].

Endocrine therapy resistant metastases can grow out in various organs beyond the pleura, including solid metastasis such as the bone, brain and liver. It is important to emphasise that our observations concerning the diminished reliance on the SGK1‐NDRG1 axis in ERα‐C samples may hold relevance primarily within the context of the specific metastatic site under investigation. Consequently, further investigations encompassing samples from diverse metastatic sites are imperative to determine the universality of these resistance mechanisms in ERα‐C samples.

The development of endocrine resistance can arise either from primary resistance or be acquired over the course of treatment. The loss ERα expression in relapsed breast cancers typically arises after prolonged endocrine therapy, contributing to acquired resistance. As a result, our study's findings are most likely relevant primarily to cases involving acquired resistance.

## 5 Conclusions

Altogether, our data show that metastatic tumours that lost ERα expression do not simply adopt an ERα‐N molecular phenotype. This may have far‐reaching implications for future clinical decision‐making and emphasises the importance of deep profiling of the metastatic tumour. Furthermore, we identified downregulation of the SGK1 kinase activity in ERα‐C advanced BCa, which could be further explored for new therapeutic approaches. Further research is warranted to understand the SGK1‐NDRG1 role in BCa disease.

## Conflict of interest

The authors declare no conflict of interest.

## Author contributions

IM‐P and WZ were involved in conceptualisation; WZ was responsible for project funding. IM‐P, KS, SB, CJ, SS, RH and CBM collected and processed all the MPE samples for (phospho)proteomics and IHC analyses. MA, PJD, JS and JW scored the IHC samples. DOD performed the (phospho)proteomic analyses and initial data processing. IM‐P and SP processed and analysed the phospho(proteomics) data. IM‐P, DOD, MA and WZ wrote the manuscript with input from all co‐authors.

### Peer review

The peer review history for this article is available at https://www.webofscience.com/api/gateway/wos/peer‐review/10.1002/1878‐0261.13540.

## Supporting information


**Fig. S1.** (Phospho)‐proteomics workflow and characteristics of proteomics data set. A. Schematic workflow of phospho‐proteomic analyses. Protein extraction and digestion was performed, followed by mass spectrometry analysis using data‐independent acquisition (DIA) mode. Spectra were matched against an in‐house library created from sample pooling, high pH fractionation and data‐dependent acquisition (DDA). For the phosphoproteomics data set, a phosphopeptide enrichment step was added. B. Number of quantified proteins per sample in the total proteomics data set. C. Number of quantified proteins per sample in the phospho‐proteomics data set.

**Fig. S2.** Distinct proteomic signature indicative of immune cell contamination. A. Unsupervised hierarchical clustering of all proteins in data set groups samples based on tumour cell percentage. The column colour bars indicate the patient group (ERα‐C (converted), ERα‐P (positive), ERα‐N (negative)), whether samples are repeat collections and tumour percentage. B. Principle Component Analyses groups samples with the lowest tumour cell content in the second component (coloured in yellow). C. Unsupervised clustering of immune cell signature proteins (derived from the ImSIg database) grouped metastatic breast cancer samples with low tumour cell content together. These samples show high abundance of immune cell proteins. The column colour bars indicate the patient group (ERα‐C, ERα‐P and ERα‐N), whether samples are repeat collections and tumour percentage. D. PCA plot showing samples that were excluded for downstream analyses (coloured in red). E. Immunohistochemistry staining for CD4, CD8, CD20 and CD68. Samples excluded or not from downstream analyses are indicated with a filled and empty dot, respectively. t‐Test was performed.

**Fig. S3.** Repeat collections from the same patient reveal a similar (phospho) proteomic landscape. A. Correlation plot of samples with repeat collections. Repeat collections are highlighted in the black boxes. Patient names are coloured by ERα‐status. Samples 6A and 6B are excluded from this as 6B had immune infiltration. B. Principle Component Analysis plot showing grouping of patients after excluding samples with low immune infiltration. Samples are coloured based on group (ERα‐C (converted) = blue, ERα‐P (positive) = red, ERα‐N (negative) = grey) C. Phospho‐proteomics data. Correlation plots of individual patients demonstrating a higher level of correlation for samples from the same patient, relative to interpatient analyses. Pearson's correlation is provided.

**Fig. S4.** Characterisation of (phospho)proteomic data. A. Principle Component Analysis plot of phospho‐proteomic samples after excluding samples with low immune infiltration. Samples are coloured by group (ERα‐C (converted) = blue, ERα‐P (positive) = red). B. Phospho‐proteomics data. Correlation plots of individual patients showing a higher enrichment of pNRDG1 peptides in ERα‐P samples than ERα‐C. Pearson correlation is provided.

**Table S1.** Clinopathological data for all pleural effusion samples. For all three patient groups (ERα‐C (converted), ERα‐P (positive), ERα‐N (negative)), relevant pathological information, patient follow‐up and treatment history is provided.

**Table S2.** Proteomics data. Missing‐forest imputated data on all proteomics samples (noncentred) used in this study, for ERα‐C (converted), ERα‐P (positive) and ERα‐N (negative) pleural effusions.

**Table S3.** Phospho‐proteomics data. Missing‐forest imputated data on all phospho‐proteomics samples (noncentred) used in this study, for ERα‐C (converted) and ERα‐P (positive) pleural effusions.

## Data Availability

Proteomics (Table S2) and phosphoproteomics (Table S3) for all tumour samples is provided in the [Link], [Link], [Link], [Link], [Link], [Link], [Link] section.
