# Supplementary figures and images for: Proteomics on malignant pleural effusions reveals ERα loss in metastatic breast cancer associates with SGK1–NDRG1 deregulation
Source: Mol Oncol. 2023 Nov 2;18(1):156–69. doi: 10.1002/1878-0261.13540 (PMC10766196; doi:10.1002/1878-0261.13540)

Supp. Fig 1

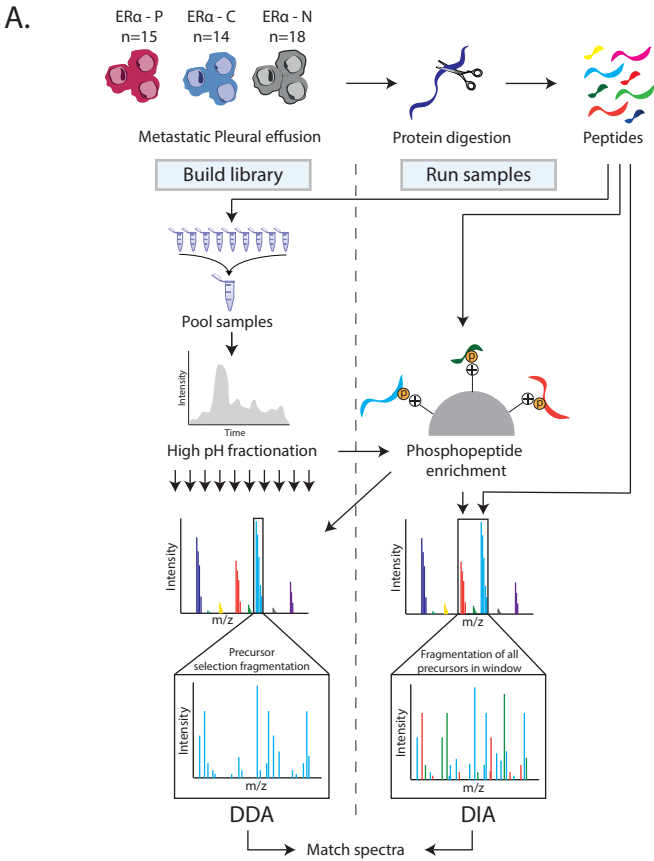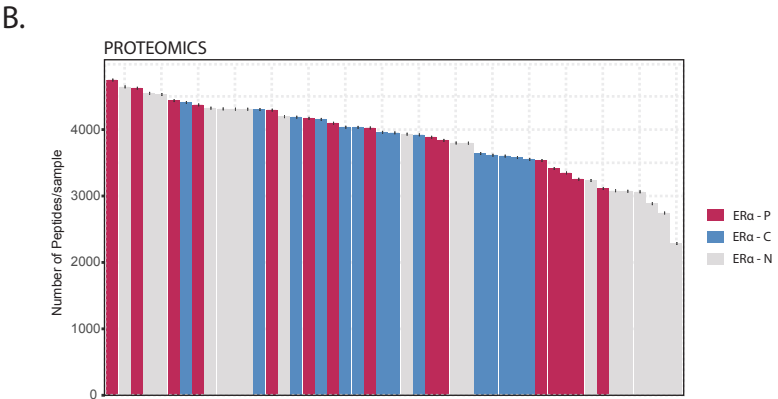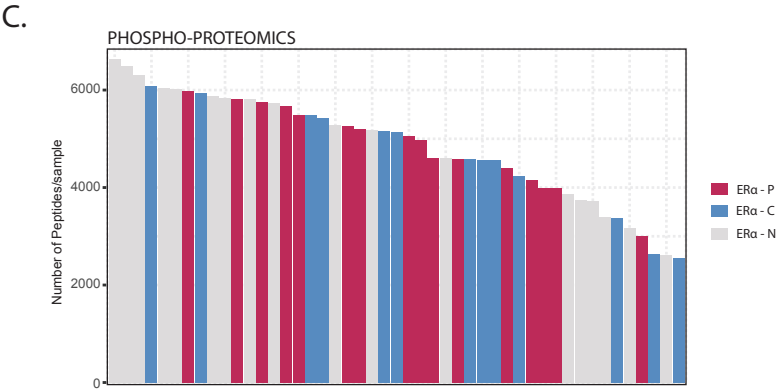

Supplement: Supplementary file 1 — Fig. S1. (Phospho)‐proteomics workflow and characteristics of proteomics data set. A. Schematic workflow of phospho‐proteomic analyses. Protein extraction and digestion was performed, followed by mass spectrometry analysis using data‐independent acquisition (DIA) mode. Spectra were matched against an in‐house library created from sample pooling, high pH fractionation and data‐dependent acquisition (DDA). For the phosphoproteomics data set, a phosphopeptide enrichment step was added. B. Number of quantified proteins per sample in the total proteomics data set. C. Number of quantified proteins per sample in the phospho‐proteomics data set. [file MOL2-18-156-s003.pdf]

Supp. Fig 2

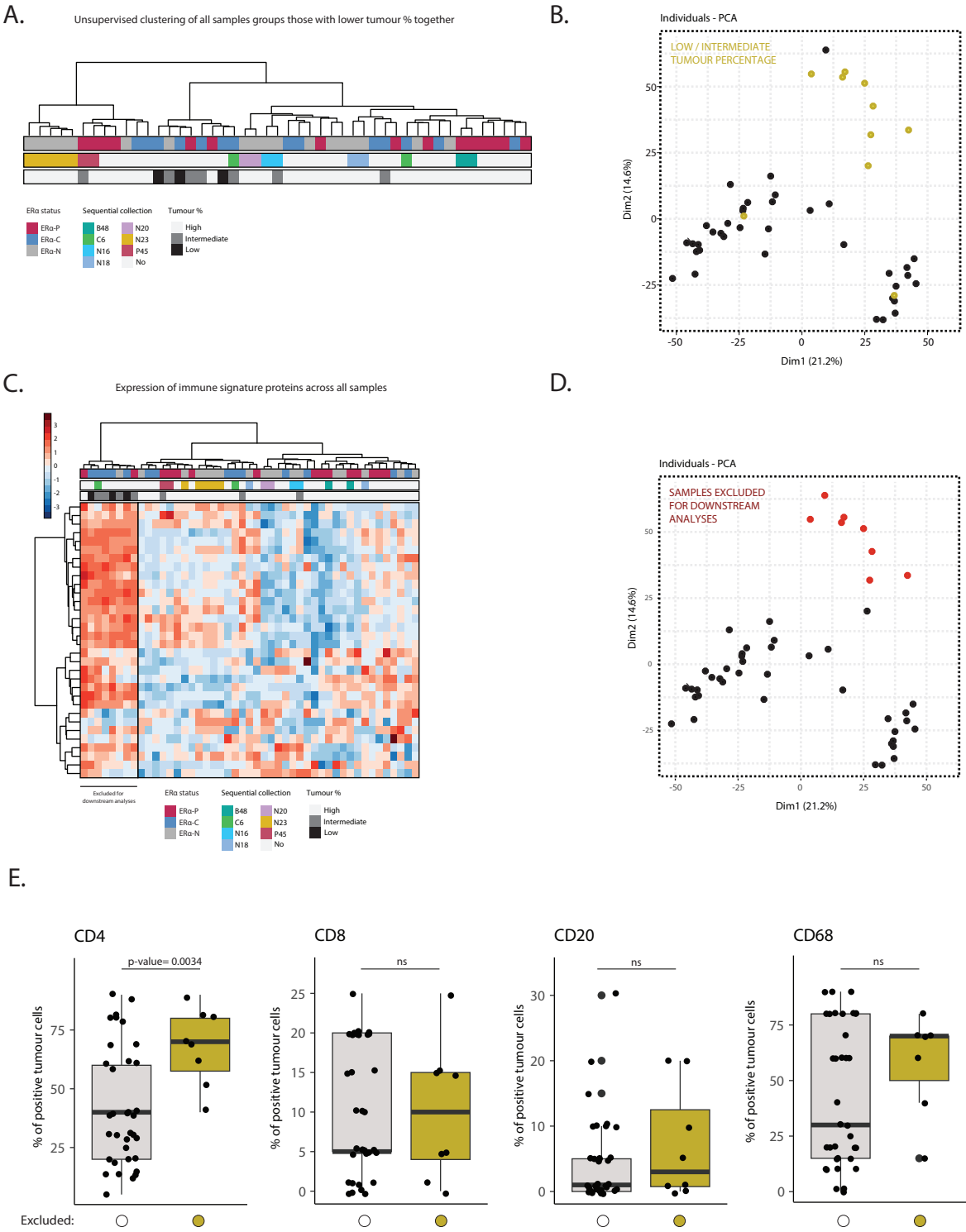

Supplement: Supplementary file 2 — Fig. S2. Distinct proteomic signature indicative of immune cell contamination. A. Unsupervised hierarchical clustering of all proteins in data set groups samples based on tumour cell percentage. The column colour bars indicate the patient group (ERα‐C (converted), ERα‐P (positive), ERα‐N (negative)), whether samples are repeat collections and tumour percentage. B. Principle Component Analyses groups samples with the lowest tumour cell content in the second component (coloured in yellow). C. Unsupervised clustering of immune cell signature proteins (derived from the ImSIg database) grouped metastatic breast cancer samples with low tumour cell content together. These samples show high abundance of immune cell proteins. The column colour bars indicate the patient group (ERα‐C, ERα‐P and ERα‐N), whether samples are repeat collections and tumour percentage. D. PCA plot showing samples that were excluded for downstream analyses (coloured in red). E. Immunohistochemistry staining for CD4, CD8, CD20 and CD68. Samples excluded or not from downstream analyses are indicated with a filled and empty dot, respectively. t‐Test was performed. [file MOL2-18-156-s005.pdf]

Supp. Fig 3

A.

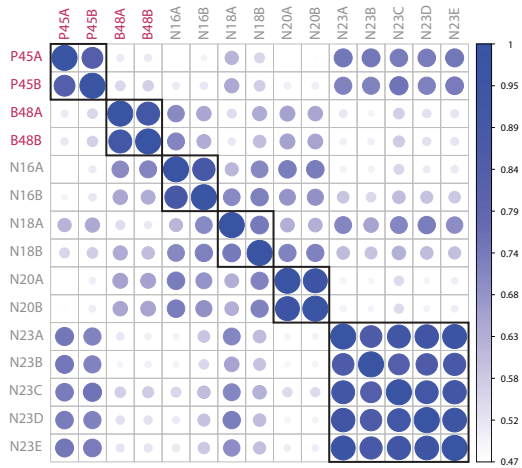

B.

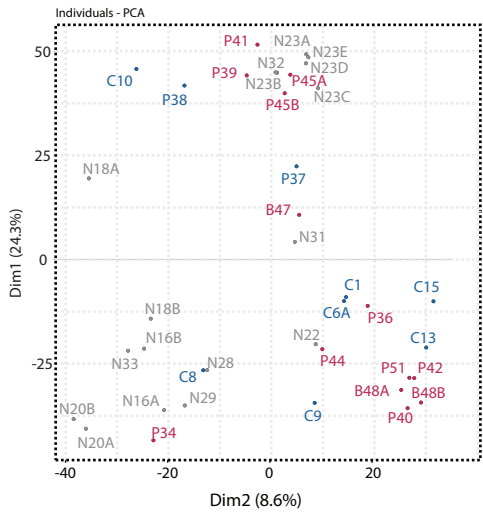

C.

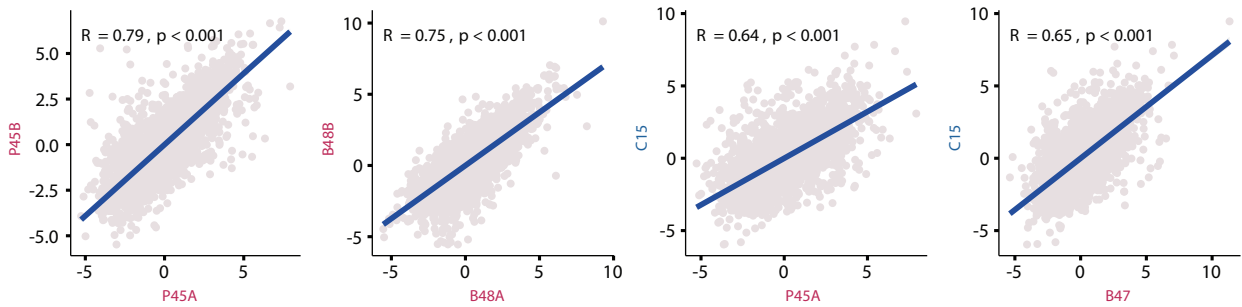

Supplement: Supplementary file 3 — Fig. S3. Repeat collections from the same patient reveal a similar (phospho) proteomic landscape. A. Correlation plot of samples with repeat collections. Repeat collections are highlighted in the black boxes. Patient names are coloured by ERα‐status. Samples 6A and 6B are excluded from this as 6B had immune infiltration. B. Principle Component Analysis plot showing grouping of patients after excluding samples with low immune infiltration. Samples are coloured based on group (ERα‐C (converted) = blue, ERα‐P (positive) = red, ERα‐N (negative) = grey) C. Phospho‐proteomics data. Correlation plots of individual patients demonstrating a higher level of correlation for samples from the same patient, relative to interpatient analyses. Pearson's correlation is provided. [file MOL2-18-156-s001.pdf]

Supp. Fig 4

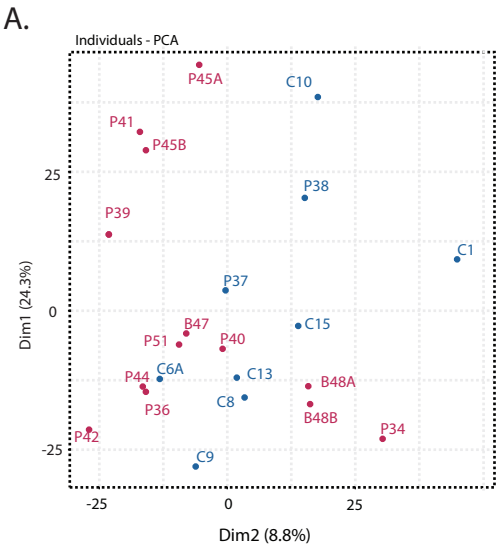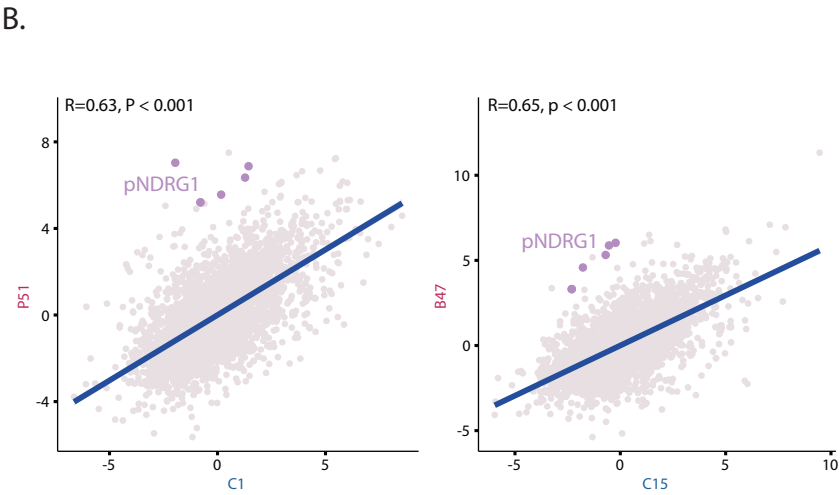

Supplement: Supplementary file 4 — Fig. S4. Characterisation of (phospho)proteomic data. A. Principle Component Analysis plot of phospho‐proteomic samples after excluding samples with low immune infiltration. Samples are coloured by group (ERα‐C (converted) = blue, ERα‐P (positive) = red). B. Phospho‐proteomics data. Correlation plots of individual patients showing a higher enrichment of pNRDG1 peptides in ERα‐P samples than ERα‐C. Pearson correlation is provided. [file MOL2-18-156-s004.pdf]
